# Virtual Health Assistants in Preventive Cancer Care Communication: Systematic Review

**DOI:** 10.2196/73616

**Published:** 2025-09-15

**Authors:** Aantaki Raisa, Xiaobei Chen, Emma G Bryan, Carma L Bylund, Jordan M Alpert, Benjamin Lok, Carla L Fisher, Lyndsey Thomas, Janice L Krieger

**Affiliations:** 1 Department of Surgery, Division of Public Health, Washington University in St. Louis, 600 S Taylor Avenue, St. Louis, MO, 63110, United States, 1 6463452302; 2 College of Journalism and Communications, University of Florida, Gainesville, FL, United States; 3 Department of Psychology, College of Arts and Science, University of Miami, Coral Gables, FL, United States; 4 Department of Health Outcomes and Biomedical Informatics, College of Medicine, University of Florida, Gainesville, FL, United States; 5 Cleveland Clinic, Lerner College of Medicine, Cleveland, OH, United States; 6 Department of Computer and Information Science and Engineering, Herbert Wertheim College of Engineering, University of Florida, Gainesville, FL, United States; 7 Lake Erie College of Osteopathic Medicine, College of Osteopathic Medicine, Bradenton, FL, United States; 8 Division of Epidemiology, Department of Quantitative Health Sciences, Mayo Clinic, Jacksonville, FL, United States

**Keywords:** virtual health assistants, cancer prevention, eHealth, intervention development, audience-centered intervention development, audience-centered design strategies, systematic review

## Abstract

**Background:**

Virtual health assistants (VHAs), interactive digital programs that emulate human communication, are being increasingly used in health care to improve patient education and care and to reduce the burden on health care providers. VHAs have the potential to promote cancer equity through facilitating patient engagement, providing round-the-clock access to information, and reducing language barriers. However, it is unclear to what extent audience-centeredness is being considered in the development of cancer-related applications.

**Objective:**

This systematic review identifies and synthesizes strategies used to make VHA-based cancer prevention and screening interventions audience-centered.

**Methods:**

Following PRISMA (Preferred Reporting Items for Systematic Reviews and Meta-Analyses) 2020 guidelines, we searched 4 databases (PubMed, Embase, Web of Science, and EBSCOhost) for peer-reviewed studies on VHA interventions promoting cancer screening (January 2022). Included studies focused on adult populations in primary care settings, with interventions emphasizing interactivity and immediacy (key VHA features). Excluded studies were on cancer treatment, noninteractive decision aids, or technical VHA development. Screening, data extraction, and quality assessment (Mixed Methods Appraisal Tool) were performed independently by multiple reviewers. Thematic synthesis was used to analyze audience-centered strategies.

**Results:**

Of 1055 records screened, 17 studies met inclusion criteria. Most (n=11) targeted colorectal cancer, with others addressing prostate, breast, cervical, or lung cancer. A total of 16 studies were US-based; 1 study focused on Uganda. Key strategies for audience-centered design included: (1) Demographic Concordance: Race or gender alignment between VHA and users (eg, African American participants interacting with Black-coded avatars); (2) User Feedback: Iterative testing via interviews, think-aloud protocols, or pilot studies to refine interventions; (3) Preintervention Needs Assessment: Identifying cultural, linguistic, or literacy barriers (eg, myths about screening in Ugandan communities); (4) Theoretical Frameworks: The Health Belief Model (most common), the Modality, Agency, Interactivity, and Navigability (MAIN) model, or tailored messaging theories guided design; (5) Information Customization: Culturally adapted content (eg, Spanish-language interfaces, narratives addressing racial disparities); and (6) Feature Customization: Adjusting VHA appearance (eg, animations and fonts) based on user preferences. Notably, 7/17 studies focused on racially minoritized groups (eg, African Americans, Hispanic farmworkers), addressing systemic barriers like mistrust in health care. However, gaps persisted in intersectional tailoring (eg, rurality and income) and non-English languages (only 2/17 studies). Recruitment methods influenced diversity; community-based strategies yielded more representative samples than solely internet-based recruitment approaches.

**Conclusions:**

The systematic review identified the audience-centered development practices currently being used for VHA-based interventions in preventive cancer care. The majority of the studies included processes to diversify and segment the intended audience, focused on medically underrepresented population groups, and implemented strategies to be culturally sensitive to the population of interest. However, opportunities remain to address multidimensional inequities (eg, rural access and low literacy). Future interventions should integrate intersectional frameworks, expand language diversity, and measure social presence to enhance engagement. This review provides a roadmap for developing equity-focused eHealth tools in cancer prevention.

## Introduction

Innovative eHealth interventions address barriers to cancer screening and improve screening rates [1]. Providing patients with customized information enables barriers to be overcome. For instance, eHealth interventions enhance patients’ knowledge, increase awareness, and alleviate embarrassment or fear about the screening procedure [2-4].

eHealth interventions can be integrated into current workflows and alert patients due for screening as automated recommendations from their clinicians [5]. Moreover, eHealth interventions are typically accessed from personal phones or computers, which reduces access barriers [6].

To ensure these eHealth interventions have the intended health outcomes for their intended audiences, they need to be audience-centered [7]. We conceptualize “Audience-centered” in this study as having a specific intended audience and identifying and addressing the specific needs of that intended audience [8]. In health intervention design, such audience-centeredness can be achieved with different approaches. Interventions can have customization of their content, language, and content; such customization can also be made at a broader level, like a group of people segmented by specific demographic characteristics, or at an individual level [8-10]. Audience-centeredness can also be achieved by getting direct input or active participation of individuals from the intended audience segment to codevelop an intervention [11,12].

Notably, we avoid the terms “patient-centered” and “person-centered” in this paper, as both have established definitions in health care communication. Patient-centered communication involves understanding patients within their sociocultural contexts to reach shared decisions about problems and treatment preferences [13]. Person-centered communication similarly emphasizes relational interactions between patients and providers [14]. We elected not to use these terms because they specifically describe interpersonal communication (whether in-person or mediated) between patients and providers. By contrast, the VHAs in our study were designed to address the needs of broader population groups (eg, stratified by race, gender, or culture) rather than individual patients. Thus, “audience-centered” more precisely describes our communication framework.

Audience-centeredness considers the intended audience’s unique or specific need for information, their cultural values, health-related beliefs, health literacy level, and trusted sources of health information [15-17]. Interventions that are audience-centered, as opposed to standardized, thus address the specific needs of the intended audience, such as their beliefs, habits, or goals [18]. For example, customizing the content of the intervention message (eg, information, language, and framing of the message) or the look and feel of the intervention delivery platform (eg, imagery, color scheme, and font) according to the intended audience’s specific needs has been shown to help reduce breast cancer survivors’ fear of recurrence compared to standardized care [19].

On the other hand, eHealth interventions designed without an audience-centered approach, that is, without audience segmentation or without identifying the specific needs of different groups, may have unintended consequences [20]. For example, an mHealth intervention promoting contraceptives among women in a collectivist society in the Global South that was developed without understanding the cultural context of the society (eg, gender relations, familial relations, beliefs about women using contraceptives, participant safety and privacy, and literacy about contraceptives) ended up increasing reported domestic violence by the participants who were in the intervention group [21]. A lack of audience-centeredness may not result in such violent consequences all the time, but it may increase the digital divide if developed without evaluating the eHealth literacy or internet accessibility of the intended audience [22]. This is especially true for medically underserved ethnic populations because mHealth interventions are predominantly developed in English and with Western or White cultural values, which creates barriers to comprehending or accepting the intervention’s messaging for individuals who speak languages other than English or have Eastern, African, or Indigenous cultural values [23].

Virtual health assistants (VHAs) are one of the emerging mHealth technologies that are being increasingly used for numerous health issues, including cancer prevention and screening. VHAs are defined as digital assistants that emulate human communication [24]. Human communication, in turn, is defined as having the quality of interactivity and immediacy [25,26]. Use of VHAs is increasing in cancer prevention because of their interactivity and engagement, ability to provide customized information, and accessibility [27]. VHAs are also being used as decision aids to help increase informed and shared decision-making regarding cancer screening [28]. However, this being a new technology, it is important to understand and identify whether interventions using this technology are being developed with audience-centered approaches due to the previously discussed importance of audience-centered intervention development. We focus on cancer prevention and screening because a lack of audience-centered communication can drastically change the outcome of a patient’s decision to screen or adhere to clinician recommendations and result in adverse health outcomes [18]. We conducted a mixed methods systematic review to identify the audience-centered approaches used to develop interactive eHealth interventions, VHAs, for cancer screening and prevention promotions. We selected a mixed methods systematic review because our goal was to include studies with diverse methodologies (eg, qualitative, quantitative, and mixed method studies)—consistent with the definition of a mixed methods review as one that incorporates multiple study designs [29]. The research question that this systematic review aims to answer is “What strategies have been used to make VHA-based cancer prevention and screening interventions audience-centered?”

## Methods

### Overview

The current systematic review thematically synthesized relevant published, peer-reviewed studies to identify audience-centered approaches applied in VHA-based intervention development. We selected thematic synthesis as our synthesis method because it allows for integrating findings across different study types (eg, qualitative, quantitative, and mixed methods studies) [30,31]. The PRISMA (Preferred Reporting Items for Systematic Reviews and Meta-Analyses) guidelines provide international standards for reporting systematic reviews. This review followed the PRISMA 2020 checklist for systematic reviews [32] for its methodology. The following methodological steps were taken to identify, screen, assess, and synthesize relevant literature for this review.

### Inclusion Criteria

Studies were included if they were primary research, published in a peer-reviewed journal, and written or available in English. All studies were required to contain research regarding the development or evaluation or both of VHA to promote cancer prevention or screening or both among adult populations in a primary care setting. We also included those studies that focused on the audience-facing aspects of the intervention, for example, VHA-audience interaction, message content, look, and feel of the intervention.

### Exclusion Criteria

Studies were excluded if their sample focused exclusively on cancer treatment and survivorship or on the technological development of the VHA (ie, algorithms, programming language, and software development). Studies were also excluded if they focused on primary prevention mechanisms, such as smoking cessation without explicitly mentioning cancer prevention as an outcome. In addition, studies were excluded if the full texts were not available in English, the studies were literature reviews themselves, were not published in peer-reviewed academic journals, or were editorials or commentaries.

### Information Sources

We searched 4 electronic databases between January 12, 2022, and January 14, 2022: MEDLINE (PubMed), Academic Search Premier (EBSCOhost), Embase (Embase), and Web of Science (Web of Science).

### Search Strategy

The search strategy was developed collaboratively by the authors (including 1 systematic review expert, CLB and 1 VHA specialist, BL) through identification of key concepts. Synonyms for these concepts were identified by reviewing highly cited literature, examining search terms from prior relevant reviews, considering database suggestions, and leveraging the authors’ and colleagues’ field expertise [27,33,34]. The complete search strategy is available in the Multimedia Appendix 1. The generic search query was as follows: (“virtual health assistant*” or Clinical Decision Support System or embodied conversational agent*)—AND (cancer* or neoplasm* or early detection or education or awareness or information) AND (prevention or screening) (The * or asterisks symbol is a commonly used wildcard symbol that broadens a search by finding words that start with the same letters preceding the symbol). Medical Subject Heading (MeSH) terms for the MEDLINE search on PubMed were acquired through rigorous MeSH library searches for cancer prevention and VHA. Similarly, Emtree terms on Embase were acquired using the built-in Emtree library.

### Selection Process

Search results from each of the 4 databases were imported to the systematic review management software Covidence (Veritas Health Innovation Ltd [35], where duplicates were automatically removed by the software. A review team was set up so that each study was reviewed by 2 researchers, and any conflict over the decision was resolved by a third researcher.

The review process was conducted in two stages: (1) title and abstract screening and (2) full-text screening. The first author (AR) trained the coscreeners (XC, EGB, and LT) with the definitions of each term and the inclusion and exclusion criteria. The coscreeners reviewed the definitions and the inclusion and exclusion criteria and provided feedback on confusing terms and processes. Based on their feedback, we revised the verbiage of definitions and inclusion and exclusion criteria on Covidence. As a group, we then practice-screened 5 titles, resolved conflicts through discussion, and further revised the definitions and inclusion and exclusion criteria. Notably, 3 screeners (2 screeners and 1 tiebreaker) completed the abstract and title screening within 3 months.

For the second review step, full-text screening, AR created a hierarchy-based list of exclusion criteria on Covidence [36]. As in the first step, 2 researchers (AR and XC) screened each full-text study to make inclusion or exclusion decisions, and a third researcher (EB) acted as a tiebreaker if there were any conflicts.

### Data Collection Process and Data Items

For data collection, we developed a preliminary data extraction template in Covidence, aligned with our research question and standard guidelines [37]. To refine this template, the first 2 authors (AR and XC) piloted it on 3 studies[38,39,40] representing different methodologies (quantitative, qualitative, and mixed methods). After comparing their extractions, they revised the template to better focus on the research question. Both authors then independently extracted data from the remaining studies, reconciling findings through weekly discussions. Each of us then independently extracted each study and consolidated the data through weekly discussions. We collected bibliographic details (eg, author, publication date, and journal), sample characteristics, VHA features, needs identified (from “Introduction,” “Result,” or “Discussion”), and needs addressed (from “Results” or “Discussion”). When information wasn’t explicitly stated, we conducted thorough reviews before coding items as “unavailable.” The complete data dictionary appears in the Multimedia Appendix 1.

### Study Risk of Bias Assessment

For quality assessment, we used the mixed methods appraisal tool [41], as it allows an appraisal of qualitative, quantitative, and mixed methods studies. Two researchers independently appraised each study, and consensus was reached upon discussion. Each study was assessed using 5 criteria (Table 1) drawn from the method used in Hong et al’s [41] study. These evaluation criteria were used to score each study, rating a study with 5 stars when 100% of the quality criteria are met, 4 stars when 80% of the quality criteria are met, 3 stars when 60% of the quality criteria are met, 2 stars when 40% of the quality criteria are met, and 1 star when 20% quality criteria are met (see Multimedia Appendix 1 for details).

**Table 1.** Bibliographic information and study characteristics.

Study ID	Journal	Cancer	Study purpose	Method	Sample size	Main findings	Quality^a^
Menon et al [40]	*Medical Care*	CRC^b^	Develop and test a theory-based computer program to boost CRC screening.	Mixed methods: focus groups and survey	199	Pilot test interventions that address doctor recommendations and fears.	3
Allen et al [42]	*American Journal of Men’s Health*	Prostate	Test a computerized-tailored decision aid to increase prostate cancer screening among African American men.	One-group pre- and post-test quasi-experiment	108	Knowledge and confidence improved significantly; significant effects were only seen in low-income men for decision-making change	3
Mosen et al [38]	*Medical Care*	CRC	Determine the effect of an automated telephone intervention on completion of FOBT^c^.	RCT^d^	5905	CRC screening increased most among 71-80-year-olds versus 51-60-year-olds postintervention.	3
Rawl et al [43]	*Health Education Research*	CRC	Compare tailored digital versus print intervention effects on CRC knowledge or beliefs at 1-week postintervention	RCT	556	The intervention group showed improved risk perception, reduced barriers, increased screening benefits, and greater knowledge, but no self-efficacy difference versus control.	3
Wu et al [44]	*2014 IEEE Systems and Information Engineering Design Symposium*	Cervical	Develop a Spanish VPE^e^ app to boost PN^f^-led cervical screening for rural Hispanic women	Survey	N/A^g^	Enhance VPE: Increase smiles, use comfier furniture, and replace paintings with plants for relaxation.	3
Krist et al [45]	*Annals of Family Medicine*	CRC, breast, and prostate	Evaluate whether patients and clinicians will use a technology-based decision module and its impact on care, using 3 cancer screening decisions as test cases.	Pragmatic observational cohort study	11,458	Boosting intervention uptake remains key; visit-linked invitations improved responses, with the greatest benefits for men, African Americans, and prostate cancer screeners.	5
Champion et al [46]	*Journal of Cancer Epidemiology, Biomarkers, and Prevention*	CRC	Compare web or phone or web+phone CRC screening messages versus usual care on completion and screening intention	Prospective randomized factorial design	1196	Phone interventions showed the highest adherence, outperforming tailored web versions, which matched usual care in stool testing, with tailoring proving inconsequential for outcomes	3
Owens et al [47]	*American Journal of Health Promotion*	Prostate	Evaluate the effects of iDecide (University of South Carolina) on prostate cancer knowledge, informed decision-making self-efficacy, technology use self-efficacy, and intention to engage in informed decision-making among African American men.	Nonrandomized controlled trial, 1-group posttest only	354	Intervention improved knowledge, decision-making, and technology self-efficacy, but benefits were greater among previously screened men	5
Griffin et al [48]	*JMIR Human Factors*	CRC	Present a case study on the design and development process for an eHealth app that uses virtual human technology to encourage CRC screening among patients aged 50 years and older.	Multimethod design: interviews, focus groups, think-aloud, participant observation, survey	354	User-centered VHAs^h^ need iterative feedback, multidisciplinary expertise, and credibility through accurate content, institutional backing, and professional design	5
Carter-Harris et al [49]	*Journal of Medical Internet Research*	Lung	Estimate the effects of a computer-tailored decision support tool that meets the certification criteria of the International Patient Decision Aid Standards will prepare individuals for and support shared decision-making in lung cancer screening decisions.	Survey	60	Both groups gained knowledge (the intervention group more), with additional increases in perceived benefits and self-efficacy only in the intervention group.	2
Vilaro et al [50]	*Psycho-Oncology*	CRC	Describe participant responses to a VHA-delivered intervention promoting CRC screening with a home stool test.	Multimethod design: focus group and think-aloud interviews	53	Participants identified 26 VHA cues categorized into 4 affordances (modality, agency, interactivity, and navigability), with acceptance improving iteratively during development.	5
Zalake et al [51]	*Intell Virtual Agents*	CRC	Propose VHA design guidelines by assessing visual, medium, and linguistic impacts on CRC communication	Mixed methods: focus groups (study 1), experiment (study 2)	1473	User perceptions depended on VHA realism, role, and provider branding; CRC intentions varied significantly by intervention format.	5
Wilson-Howard et al [52]	*JMIR Formative Research*	CRC	Integrate Black men’s views to design a VC^i^ for FIT^j^ kit promotion in future CRC screening clinical trials	Focus groups	25	Men’s feedback refined the VC’s visuals and movement, while heuristics (social presence, novelty, and authority) boosted credibility, earning the trusted “brother-doctor” role, with many preferring it over physicians.	5
Vilaro et al [53]	*BMC Medical Informatics and Decision Making*	CRC	Describe specific design features (eg, cues) adapted to improve the acceptability and usability of a web-based intervention promoting CRC screening.	Multimethod design: focus groups, think-aloud interviews	53	Users prioritized VC interactivity, trust, and expertise, with iterative comments progressively refining source credibility cues	5
Krieger et al [54]	*American Journal of Preventive Medicine*	CRC	Determine the efficacy of the ALEX^k^ VHA intervention to increase patient intentions to talk to their doctor about CRC screening.	RCT	1363	Animated VHAs outperformed on discussion intentions with doctors; race concordance mattered only for static VHAs for intention to screen, alongside demographics and trust predictors	3
Kabukye et al [55]	*JMIR mHealth and uHealth*	Generic	Assess Ugandan cancer awareness, develop IVR^l^ education, and evaluate its adoption	Mixed methods: focus group, interviews, survey, cocreation workshops	113	Participants sought comprehensive cancer information. IVR familiarity was high; they praised convenience but requested live agents and better marketing	3
Vilaro et al [39]	*Health Communication*	CRC	Describe and compare perceptions of CRC screening among adults at average risk of CRC and identify appropriate strategies and messages to facilitate guideline-concordant screening.	Focus groups	154	All groups valued friendly, trustworthy VHAs. Black adults preferred CRC risk info from VHAs and sharing interventions more than Whites	5

a The number of asterisks (*) refers to Hong et al’s [41] quality evaluation criteria, which rate a study with 5 when 100% of the quality criteria are met, 4 when 80% are met, 3 when 60% are met, 2 when 40% are met, and 1 when 20% are.

b CRC: colorectal cancer.

c FOBT: fecal occult blood testing

d RCT: randomized controlled trial.

e VPE: Virtual patient educator.

f PN: patient navigator.

g Not available.

h VHA: virtual health assistant.

i VC: virtual clinician.

j FIT: fecal immunochemical test.

k ALEX: Agent Leveraging Empathy for eXams.

l IVR: Interactive voice response.

### Synthesis Methods

Using our standardized template, we exported all extracted data to Excel (Microsoft Corp) for analysis. First, we synthesized VHA intervention characteristics across studies (Table 2) and participant demographics (Table 3), comparing them with each intervention’s intended audience. For thematic synthesis [30], we examined the “Introduction,” “Methods,” “Results,” and “Discussion” sections to identify audience-centered strategies (Table 4). The first author conducted initial coding of “needs identified” and “needs addressed” text segments, creating descriptive labels for audience-centered approaches. These codes were then collaboratively reviewed by the first 2 authors and grouped into thematic strategies through an iterative process, with final themes corresponding to those presented in Table 4

**Table 2.** Virtual health assistant characteristics of the interventions.

Study ID	Intervention name	VHA^b^ purpose	VHA modality	VHA appearance	Participant input method
Menon et al [40]	TIMS^c^	Increase CRC^d^ screening.	Text	N/A^e^	Touchscreen
Allen et al [42]	Decision aid	Promote informed decision-making for prostate cancer	Visual, voice	N/A	Touchscreen
Mosen et al [38]	Automated call	Induce completion of CRC screening.	Voice	N/A	Touchscreen or dial button
Rawl et al [43]	Colon Testing: Celebrate Life for Years to Come	Improve CRC-related knowledge and health beliefs.	Visual, voice, text	Black men and women in social and clinical settings emulating real-life experiences of Black Americans and the process of CRC screening	Click or touchscreen
Wu et al [44]	ECA^f^	Increase CRC screening rate.	Visual, voice, text	An animated, professionally attired Hispanic woman sitting in a generic setting	Click or touchscreen
Krist et al [45]	Informed decision-making module	Aid decision-making for 3 cancers.	Visual, text	Interactive web page with tailored images and options	Click or touchscreen
Champion et al [46]	Tailored web-based intervention	Increase completion of CRC screening.	Visual, voice, text	N/A	Click or touchscreen
Owens et al [47]	iDecide	Promote informed decision-making about prostate cancer screening.	Visual, voice, text	An animated Black man, wearing a white coat like a clinician in an office-like environment (with computer desks and computers)	Touchscreen
Griffin et al [48]	Agent Leveraging Empathy for eXams (ALEX)	Encourage CRC screening.	Visual, voice, text	N/A	N/A
Carter-Harris et al [49]	LungTalk	Prepare individuals and support shared decision-making for lung cancer screening.	Visual, voice, text	N/A	Click or touchscreen
Vilaro et al [50]	VHA	Promote CRC screening using home stool test.	Visual, voice, text	Variations in doctor-looking VHAs (in white coats or in scrubs) were tested among the target audience	N/A
Zalake et al [51]	ALEX	Deliver information related to CRC.	Visual, voice, text	Doctor-looking male and female Black and White animated characters in white coats, in a hospital-looking room	Touch or tap or click
Wilson-Howard et al [52]	ALEX	Deliver precision messages promoting the FIT^g^ kit for CRC screening.	Visual, voice, text	Black male animated character in different poses and settings	N/A
Vilaro et al [53]	VHA	Promote CRC screening.	N/A	Black female animated character in a doctor’s white coat in different settings	N/A
Krieger et al [54]	ALEX	Deliver the CRC screening message.	Visual, voice, text	Black and White male and female animated characters in a hospital-like setting wearing white coats	N/A
Kobukye et al [55]	IVR^h^	Deliver a cancer awareness message.	Voice	N/A	Touchscreen
Vilaro et al [39]	Meet ALEX	Embody culturally sensitive CRC screening message.	Visual, voice, text	N/A	N/A

b VHA: virtual health assistant.

c TIMS: Tailored Intervention Messaging System.

d CRC: colorectal cancer.

e Not available.

f ECA: embodied conversational agent.

g FIT: fecal immunochemical test.

h IVR: interactive voice response.

**Table 3.** Audience characteristics of the interventions.

Study ID	Intended audience	Sample race	Sample age (years)	Sample gender	Sample education	Sample income	Justification for using VHA^a^
Menon et al [40]	Average-risk patients in primary care clinics	Black or African American (78/101, 52.3%, other (23/101, 46%)	55-64	Men and women	Secondary^b^ and higher^c^ education	Low	Tailored health education improves health behavior.
Allen et al [42]	African American men	Black or African American	more than 40	Men	Secondary and higher education	Low to middle	Accessibility Modifiability Lack of need for communication with a doctor Usability in nonclinical settings
Mosen et al [38]	Average-risk patients who are out of the guideline for screening	White or European American (2729/2943, 92%), other or unknown (214/2943, 8%)	40-64	Women	N/A^d^	N/A	Automated reminder calls can improve adherence to screening while being cost-effective compared to live human agent calls.
Rawl et al [43]	Average-risk African American men and women	Black or African American	55-64	Men and women	Secondary education	Low^e^	Increasing use of eHealth interventions.
Wu et al [44]	Hispanic farmworkers in Dover, Florida	Latina	N/A	Women	N/A	N/A	Effectiveness of interactive multimedia interventions among the target audience. Effectiveness of tailored digital communication to enhance and supplement patient-clinician communication among the target audience.
Krist et al [45]	Patients who are likely to be making decisions for cancer screening	Asian American (60/903, 6.6%), Black or African American (63/903, 7%), White or European American (618/903, 68.4%)	40-54	Men and women	N/A	N/A	Effectiveness of decision aids and digital health technologies in systematically automating decision-making processes outside the constraints of clinical encounters.
Champion et al [46]	Average-risk American women who are out of guideline for screening	Asian American or Pacific Islander or other (40/1196, 3.4%), Black or African American (124/1196, 10.4%), White or European American (1032/1196, 86.3%)	55-64	Women	Higher education	Low to middle	Customizability of VHAs. Effectiveness of tailored interventions to improve health behavior.
Owens et al [47]	African American men	Black or African American	55-64	Men	Secondary and higher education	Low to middle	VHAs incorporated with decision aids have been proven an effective technique for promoting knowledge and behavior change, as it uses human-like interactivity. Pilot research showed that the target audience was open to the intervention.
Griffin et al [48]	Average-risk Americans aged 50 years or older	N/A	more than 40	Men and women	N/A	N/A	Focus group discussions showed that participants were open to discussing their health with a VHA. VHA provides a sense of anonymity to discuss sensitive health issues. Tailored information improves health behavior change. Demographically concordant care can address poor health outcomes seen in demographically discordant care.
Carter-Harris et al [49]	Individuals eligible for cancer screening	Black or African American (10/60, 17%), White or European American (48/60, 80%), Other (2/60, 2%)	55-74	Men and women	Secondary and higher education	Low to middle	The lung cancer screening rate is low. Discussion with clinicians is important for high-risk individuals (smokers or former smokers). An interactive decision aid tailored to individuals’ varying needs can help discussion with clinicians.
Vilaro et al [50]	Black adults	Black or African American	more than 40	Women	N/A	N/A	VHAs can be culturally tailored and can help disclose sensitive information, especially among minoritized groups who have stigma and fear of being judged by health care providers.
Zalake et al [51]	Not specified	Black or African American (319/1473, 21.7%), White or European American (1152/1473, 78.2%), Other (2/1473, 0.1%)	more than 40	Men and women	N/A	N/A	VHAs combine the benefits of digital intervention (ie, low costs) and interpersonal communication.
Wilson-Howard et al [52]	African American men	Black or African American	more than 40	Men	Secondary and higher education	Low	eHealth interventions can be low cost, easier to disseminate, and provide modifiable or personalized health information in real time (no specific benefits of VHAs mentioned).
Vilaro et al [53]	Older, racially minoritized patients	Black or African American	more than 40	Women	None to secondary education	Low to middle^f^	Not specified
Krieger et al [54]	Adults eligible for CRC^g^ screening	White or European American (1076/1363, 78.9%), Black or African American (287/1362, 21.1%)	55-64	Men and women	N/A	N/A	COVID-19 presented an opportunity to leverage technology to improve screening adherence. VHAs can be easily tailored to patient preferences. VHAs provide better accessibility.
Vilaro et al [39]	Rural Black and White adults	Black or African American (85/154, 55.2%), White or European American (69/154, 44.8%)	more than 40	Men and women	Higher education	Low	VHAs can be customized to provide racially concordant care.
Kabukye et al [55]	General Ugandan population	N/A	18-39	Men and women	None to higher education	N/A	eHealth is cost-effective, and especially beneficial in low- and middle-income countries to improve health communication, education, and awareness of chronic illness. eHealth addresses access and cost barriers.

a VHA: virtual health assistant.

b secondary education (grades 7-12).

c higher education (post grade 12).

d N/A: not available.

e low income (less than US $52,000/year).

f middle income (US $52,000-US $156,000/year).

g CRC: colorectal cancer.

**Table 4.** Strategies used to make the virtual health assistant–based interventions patient-centered.

Study ID	Race gender concordance	User feedback	Preintervention needs exploration	Theoretical framework	Message customization	VHA^a^ features customization
Menon et al [40]	✓	Focus group feedback is used to customize intervention. An RCT^b^ is used to test intervention	Cost-effective strategies to provide integrated, accessible health care services in practical settings	Health belief model	N/A	Navigation, color, font, and graphics, cultural sensitivity to the intended audience
Allen et al [42]	✓	Pre and posttest quasi-experiment was used to test the effect of the intervention	Black^c^ adults are 60% more likely to develop CaP^d^ and 2.4 times more likely to die from it. Most interventions for CaP screening among Black men have been educational as opposed to promoting IDM^e^	Ottawa Decision Support Framework	Personal risk assessment	Audiovisual cues that are culturally appropriate
Mosen et al [38]	✓	Users tested the intervention through RCT	Low uptake of CRC^f^ screening among eligible adults compared to other cancer screening rates	N/A	Reminder calls to participants who requested a screening kit but had not returned a stool sample	N/A
Rawl et al [43]	✓	A community advisory group evaluated messages and provided feedback Users tested the intervention through RCT	Lack of knowledge about test options Importance of screening in the absence of symptoms Curability of CRC when detected early Lack of time, inconvenience, lack of interest, cost, fear of positive result, embarrassment, and cancer fatalism	Health belief model	Personal colon cancer risk profile based on age, gender, and family history of the participant	Racially concordant characters in video demonstration Culturally relevant family setting (birthday party of a screening-eligible person)
Wu et al [44]	✓	Participants interacted with the intervention in different phases, providing feedback for improvement	US Hispanic women are twice as likely to have cervical cancer as White^g^ women Disparities are influenced by cultural beliefs, linguistic barriers, socioeconomic status, health literacy, and provider behaviors and practice patterns that add to the disparities	N/A	Options for users to choose what kind of information they want to know more about Reduced medical jargon	Spanish language, dialect Talking speed, tone, and volume Camera angle of the VHA Ambient sound VHA appearance
Krist et al [45]	✓	Participants completed the decision module, invited to test the module over 3 phases, and provided feedback to modify the next phase	Limited support for patients during the screening decision-making process Time constraints inhibit clinicians’ ability to provide detailed information Patients want decisional support and to be included in the decision-making process for cancer screening	N/A	Risk of cancer Benefits or harms of screening, Screening logistics based on each participant’s readiness for a cancer screening decision, Fears, Desired assistance, Informational needs, Preferred format for receiving information and statistics Planned next steps	The decision module customized the page with the preferred format of each patient, for example, words, numbers, pictures, or stories
Champion et al [46]	✓	Users tested the intervention through RCT	Tailoring to demographic and belief variables (eg, perceived risk, perceived benefits, perceived barriers, self-efficacy, fatalism, and fear) increases relevance of intervention messages, thereby increasing intervention effects for various health behaviors	Health Belief Model Transtheoretical model Elaboration likelihood model	CRC risk information, benefits of screening, suggestions to overcome barriers based on participant response to perceived risk and benefits, and personal barriers–related questions	N/A
Owens et al [47]	✓	Iterative process used to develop the intervention with feedback from Black men Participants tested the intervention in a one-group pre or posttest experiment	CaP rates are highest among Black men Few studies focus on increasing CaP knowledge among Black men despite it being a critical component for IDM Racially minoritized individuals are less likely to engage in IDM partly due to lack of decision self-efficacy	Unified Theory of Acceptance and Use of Technology model Cognitive Theory of Multimedia Learning	Information about informed decision-making, including risk and benefits of screening Questions and exercises to help with information retention	Black male animated avatar providing information Buttons to click on to repeat the information
Griffin et al [48]	✓	Participants were observed while using the intervention Members of the development team were interviewed for feedback	Increased use of eHealth Common barriers to screening (eg, cost, time, and embarrassment)	Theory-based constructs	Common barriers to screening, including cost, time, and feelings of embarrassment caused by collecting a fecal sample	VHA appearance, environment, and other symbols (eg, credible logo or name)
Carter-Harris et al [49]	✓	Users tested the intervention through pilot RCT	Due to potential risks of screening using low-dose computed tomography, lung cancer screening is a preference-sensitive decision and requires patient-clinician discussion and a shared decision-making process Knowledge and awareness of lung cancer screening among the general population are extremely low	Conceptual model for lung cancer screening participation (combination of Health Belief Model and Precaution Adoption Process Model)	Lung health and screening information tailored by smoking status Offers questions the user can ask to initiate a discussion with their clinician Includes specific questions identified by the user	N/A
Vilaro et al [50]	✓	Participants tested the intervention in focus groups and think-aloud interviews	Only 8% of Black adults use FIT^h^ to screen for CRC Possible barriers include low acceptance or knowledge of the noninvasive options among both physicians and patients Black adults rely on physician recommendations for screening	Modality, Agency, Interactivity, and Navigability model Credibility	Information consistency and understandability of the message were modified based on audience feedback Participants also identified missing content and confusing information to improve message content	VHA attire, voice, talking speed modified based on participant feedback to increase trustworthiness, credibility, expertise, and social presence
Zalake et al [51]	✓	Participants provided feedback through focus groups (phase 1); and through survey (phase 2)	User perceptions of VHA (eg, likability, friendliness, and trustworthiness) are affected by change in visual design, hence appropriate VHA design is essential in medicine	N/A	N/A	Organizational branding, VHA visual framing changed based on participant feedback to increase VHA credibility and trustworthiness
Wilson-Howard et al [52]	✓	Participants provided feedback in focus groups	Multiple factors contribute to CRC inequities among Black men, including poorer access to preventive screening, aversion to colonoscopy, and limited knowledge of alternative screening modalities	Modality, Agency, Interactivity, and Navigability model	N/A	VHA modality, navigability, interactivity, and other visual perceptions adapted based on user feedback
Vilaro et al [53]	✓	Participants tested the intervention in focus groups	FIT can be a good screening option to reduce racial inequities faced by Black, rural adults VHA-based information delivery can be used to promote FIT in rural places.	Technology acceptance model, social presence	Accuracy and relevance of information adapted based on user feedback	VHA appearance, movement, attire, ambience, and navigability adapted based on user feedback
Krieger et al [54]	✓	Users tested the intervention through RCT and provided feedback through questionnaire	Patients are more likely to follow screening recommendations when delivered by a race-concordant clinician	N/A	N/A	Racial concordance between VHAs and intended users
Kabukye et al [55]	✓	Participants tested and provided feedback on the intervention in focus groups and workshops	Geographic barriers to cancer care Lack of cancer information Stigma of cancer Technology-based interventions can address accessibility issues	Health belief model Unified Theory of Acceptance and Use of Technology	Information on what cancer is, what causes cancer, cancer screening and diagnosis, and practical information on what to expect during cancer care incorporated into the intervention based on user feedback	Options for people to speak with a real live agent if preferred
Vilaro et al [39]	✓	Users tested the intervention in different phases in focus groups	Studies show that racial discordance in health care interactions, in which patient and provider perceive each other as belonging to a different race, reduces patient compliance with medical recommendations, lowers patients’ perceptions of the quality of medical care, and reduces patients’ communication satisfaction and perceptions of trust in providers	Critical race theory	N/A	VHA appearance, ambience, and voice changed to improve trustworthiness, expertise, and authority cues

a VHA: virtual health assistant.

b RCT: randomized controlled trial.

c Black: Black or African American.

d CaP: prostate cancer.

e IDM: informed decision-making.

f CRC: colorectal cancer.

g White: White or European American.

h FIT: fecal immunochemical test.

## Results

### Study Selection

**Figure 1.**
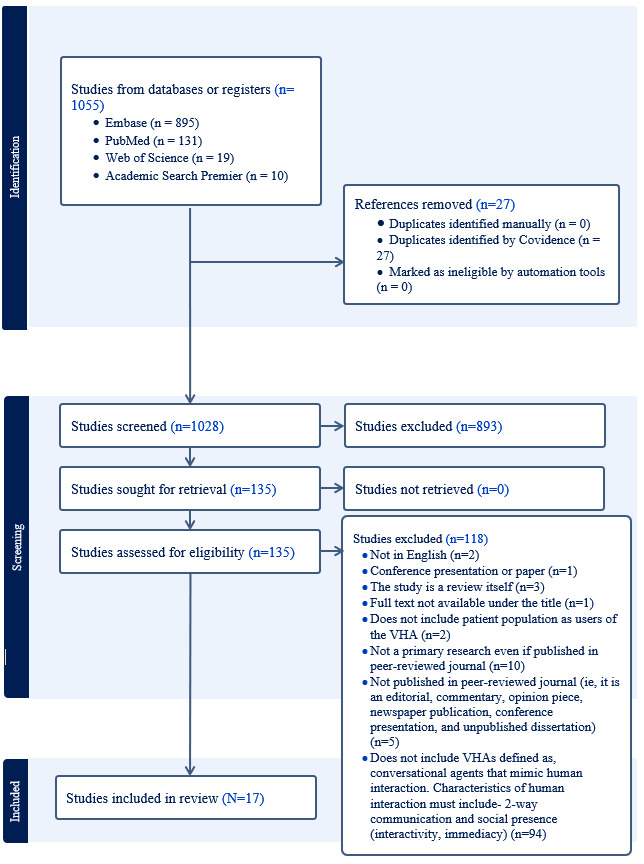
PRISMA Preferred Reporting Items for Systematic Reviews and Meta-Analyses flowchart.

A search of the 4 databases yielded 1055 results, 27 of which were removed as duplicates. During the first step of screening, title and abstract screening, 893 studies were excluded as irrelevant. In the second step, full-text screening, 118 more studies were excluded for not meeting the inclusion criteria (Figure 1). The majority of the studies during full-text screening (n=94) were excluded because they were not VHA-based interventions as defined in the inclusion criteria. For the detailed screening process, please see Figure 1.

### Study Characteristics

A total of 17 studies [38-40,42-55]. These studies were published between 2008 and 2022, with 6 [39,51,52,53,54,55] of them published since 2021 alone. Of the 17 studies [38-40,42-55] included, 9 [38,42,43,44,45,46,47,49,54] were quantitative, 3 mixed methods [40,51,55], 2 qualitative [39,52], and 3 multimethod qualitative studies [48,50,53]. The majority of the studies (n=11 [38,39,40,43,46,48,50,51,52,53,54] focused on colorectal cancer while others addressed prostate cancer (n=2 [42,47]), lung cancer (n=1 [49]), cervical cancer (n=1 [44]), combined prostate, colorectal cancer, and breast cancers (n=1 [45]) or general cancer (n=1 [55]; Table 1). Notably, 16 of the studies were conducted in the United States; 1 study [55] was conducted in Uganda. Two interventions were developed in languages other than English: one in Spanish [44] and the other in Luganda [55]. The majority of the studies (n=9 [39,40,43,45,48,49,51,54,55]) included both men and women in the sample, 3 studies [42,47,52] included men only and 5 studies [38,44,46,50,53] included women only. Around 6 studies [42,43,47,50,52,53] focused exclusively on African Americans, 1 study [44] focused on Hispanic women, and 1 study [55] focused on a Ugandan population. Two studies [39,40] had multiple races with a Black majority, 1 study [48] did not mention sample race. The remaining studies (n=6 [38,45,46,49,51,54]) had participants from multiple races and ethnicities in their samples, but predominantly European Americans (Table 3).

### Risk of Bias

Altogether, 8 [38,40,42,43,44,46,54,55] out of the 17 studies [38-40,42-55] satisfied 3 of the 5 quality assessment criteria while another 8 studies [39,45,47,48,50,51,52,53] satisfied all 5. Notably, one study [49] satisfied 2 quality assessment criteria (Table 1). All 17 studies [38-40,42-55] satisfied the 2 screening questions pertaining to having clear research questions and a data collection method appropriate for the research questions. Hence, despite the varying levels of quality, none of the studies were excluded from the synthesis (see Multimedia Appendix 1).

### Characteristics of VHAs

Studies used inconsistent definitions of Virtual Health Assistants (VHAs), and some provided no definition at all. There was no consensus on the definition of virtual assistants, or even any standard terminology, between studies conducted by different research groups. The terms used for virtual assistants differed and included embodied conversational agent, VHA, decision aid, and other generic names such as interactive web interventions or tailored web-based interventions. The modality of the interventions evolved over time, starting with text-based web interventions and expanding by adding audio or voice and visuals for participants to interact with. Three of the interventions [38,46,55] used tailored automated voice calls as part of the intervention, and all other studies were web-based (Table 2).

Common characteristics of the VHAs in these studies included being web-based, providing tailored information based on user responses to certain questions or cues, and providing some interactivity in terms of the user being able to select responses. None of the VHAs used natural language processing. All the VHAs were preprogrammed with specific options, from which the users could select the most suitable options for themselves.

The VHAs that used animated characters for communication applied appearance-based concordance with the intended audience, with either race or gender or both (Tables2  3). Notably, 8 [43,44,47,50,51,52,53,54] of the studies used animated characters to convey cancer-related information; 1 study [42] did not explicitly mention whether the characters were animated or were recordings of real humans. Specifically, 6 studies [39,50,51,52,53,54] that were all about the same intervention used an anthropomorphic name for the intervention: ALEX (Agent Leveraging Empathy for eXams) (Table 2).

### Strategies of Audience-Centered Development

Thematic synthesis of the studies resulted in 6 major strategies used to make the interventions audience-centered. These strategies can be categorized broadly into two types: (1) how the intervention was developed to be audience-centered and (2) what was customized in the interventions to make them audience-centered. The interventions were developed to be audience-centered by using 4 strategies, such as gender and race concordance, user feedback, preintervention needs exploration, and theoretical framework. Two aspects of the interventions were customized for audience-centeredness, namely information and VHA features.

#### Gender and Race Concordance

All 17 studies [38-40,42-55] recruited participants from the population groups for which the interventions were intended (Table 3). Such concordance was specifically based on race and gender. Six studies [42,43,47,50,52,53] were designed for African American adults and recruited accordingly. One study [44] recruited Hispanic farmworkers, and another study [55] recruited Ugandan adults, each in concordance with their intervention’s intended audience. Studies that targeted a general population tended to recruit mostly European (White) American participants. Although none of the studies mentioned location, education level, or income when describing their intended audience, many studies coded these characteristics for data analysis. In total, 8 [ 39,40,42,43,46,47,49,52] of the 10 studies [39,40,42,43,46,47,49,52,53,55] that reported sample education level recruited participants with higher education (ie, with college, university, or graduate degrees). Notably, 5 [42,46,47,49,53] of the 9 studies [39,40,42,43,46,47,49,52,53] that reported sample income recruited mostly low-to-middle-income participants, and the other 4 studies [39,40,43,52] recruited exclusively low-income participants. Around 8 studies [39,40,42-44,52,53,55] reported sample locations. Of these, 4 studies [40,42,43,55] were exclusively recruited from urban areas, and 4 studies [39,44,52,53] from rural areas.

#### User Feedback

Regardless of study design, all the interventions were tested among study participants to collect their feedback. Such feedback was collected to understand what needed to be changed or modified in the intervention to make it more audience-centered. The qualitative (monomethod and multimethod) studies (n=5 [39,48,50,52,53]) collected feedback with interviews, think-aloud interviews, and focus group discussions. Sample participants interacted with or used the intervention and provided feedback. Based on participant feedback, researchers recommended improvements to the intervention. In the mixed-method studies (n=3 [40,51,55]), the qualitative part of the studies had participants interact with or use the intervention and provide feedback. In the quantitative part of the mixed methods studies, the newer versions of the interventions developed from the audience feedback were tested using statistical methods to make inferences about intervention effectiveness. The quantitative studies (n=9 [38,42,43,44,45,46,47,49,54]) pilot-tested, fully tested, or observed the interventions’ effectiveness on outcome measures among participants.

#### Preintervention Needs Exploration

All the studies (n=17 [38-40,42-55]) provided a rationale for developing a cancer prevention intervention in their “Literature Review” or “Introduction” section, based on previous publications. The studies based their rationale on a lack of uptake in the relevant cancer screening among a general or specific population group. However, these rationales were not specific to VHA-based interventions but rather justified a need for any or a better intervention to promote cancer screening or awareness, or both. The most cited audience need specific to VHA interventions was the need for tailored information based on intended audiences’ preferences (n=11). Studies also cited various other benefits of eHealth interventions, including accessibility, lack of need to visit a clinical setting, and cost-effectiveness.

Benefits specific to animated VHAs included interpersonal communication and demographic concordance. The studies focusing on minoritized populations (n=7) explicitly mentioned specific barriers of accessibility, discrimination faced in the health care system, cultural values not being addressed in traditional health care settings, and lack of demographically matched clinicians.

#### Theoretical Framework

A total of 11 studies [39,40,42,43,46,47,49,50,52,53,55] explicitly mentioned using one or multiple theories, models, or frameworks (henceforth called theory) in the development of the intervention. One study [48] mentioned specific theoretical constructs instead of a single theory. The rest of the studies (n=5 [38,44,45,51,54]) did not report the use of any theory. However, each of these studies mentioned at least 1 construct drawn from established behavioral theories (eg, behavioral intention) or intervention development frameworks (eg, tailoring). In the studies that mentioned theories explicitly, the health belief model was the most frequently used (n=4 [40,46,49,55]. The second most frequently used theories were the Modality, Agency, Interactivity, and Navigability (MAIN) model and the Unified Theory of Acceptance and Use of Technology (n=2 each [50,52]. All the other theories mentioned were used in 1 study each (Table 4).

Theory was mainly applied to identify behavioral constructs that needed to be incorporated into the intervention to achieve the intended outcomes (eg, improve intention to screen). The incorporation of the theoretical constructs into the interventions was 2-fold. First, the constructs were used to develop the (verbal and nonverbal) message delivered through the intervention. Second, the constructs were used to develop the intervention platform (eg, navigation, look, and feel).

#### Information Customization

Information about cancer was the most common aspect of the VHAs that was customized to make the intervention audience-centered. Information regarding the audience’s cancer-related informational needs, screening knowledge, and concerns was incorporated into the interventions. Some of the studies specifically focused on cultural tailoring of the intervention, including surface-level tailoring, namely race and gender matching and deep-structural tailoring, such as addressing values and beliefs related to racial identity, cancer, and cancer screening, and addressing myths or stigma regarding cancer or cancer screening.

#### VHA Features Customization

Customizing VHA features based on audience input depends on the type of intervention. The VHAs in the included studies can be categorized into 4 types: text only (n=1), voice only (n=2), visual and voice and text combined (n=3) and visual, text, and voice combined (n=10). The features of the text-only and voice-only (n=3) interventions were not modified. However, the VHAs that provided information with any combination of voice, text, and visuals (n=13) were modified based on audience input. For the animated VHAs, their movements, facial expressions, voice, and settings were modified based on the audience feedback. Moreover, the animated VHA interventions’ color palettes, text size, and fonts for subtitles were also modified based on audience feedback.

## Discussion

### Principal Findings

The current systematic review identified existing practices in the development of VHA-based interventions for cancer prevention and screening. There are 4 key takeaways from this review. First, we found that there is an intentional effort to make VHAs audience-centered, as opposed to developing generic interventions without any audience segmentation. The studies included in this review focused on providing customized information regarding cancer and screening to the intended audience. The information was customized based on the specific cancer(s), individual risk of the cancer, guideline for screening, and screening methods. Some studies also customized different features of the intervention with surface-level cultural attributes, such as using race- and gender-matching pictures or animation and delivering information in the native language of the intended audience.

Second, the current review identified an emphasis on developing audience-centered eHealth interventions to address cancer screening barriers faced by racially minoritized groups in the United States. Around 6 [42,43,47,50,52,53] of the 17 studies [38-40,42-55] focused exclusively on developing or testing or both of VHA-based interventions to improve cancer screening among African Americans, and 1 study [44] focused exclusively on cancer screening among Hispanic American women. This is an important step toward reducing health disparities, since racially minoritized individuals often face discrimination in health care systems due to unconscious biases held by clinicians [56]. VHAs developed with the needs of racially minoritized individuals can address and mitigate such biases in preventative cancer care.

Third, of the 17 included studies [38-40,42-55], 12 [39,40,43-45,47,48,50-53,55] mentioned the importance of cultural sensitivity of eHealth interventions, and 3 [39,43,48] explicitly explained how culture was incorporated in the intervention. Rawl et al [43] evaluated the cultural appropriateness and relevance of the intervention through a community advisory board. Griffin et al [48] mentioned using cultural probing as a method for audience need assessment and the effectiveness of iterative testing of interventions among intended audiences to improve the cultural competency of the messages. Vilaro et al [39] included subjective culture and critical race theory to delve deeper into the structures of culture by exploring the individual and racial identities of the intended audience to explore the relationship between identity and perceptions of the VHA.

Studies that did not detail the incorporation of culture in the interventions or did not mention culture did not necessarily fail to incorporate cultural sensitivity. As mentioned, all studies had their interventions tested by members of the intended audience and incorporated this feedback into the intervention design. This process alone provides ample opportunity to incorporate participants’ cultural needs, even when culture is not explicitly mentioned [11]. For example, Kabukye et al [55], who conducted the study in Uganda to improve cancer awareness among the general Ugandan population, developed their intervention using interview and focus group data from Ugandan health care workers, cancer specialists, cancer survivors, and lay audience members without cancer to incorporate a multitude of perspectives in the message design.

Finally, this review identified certain areas of improvement for greater audience-centeredness of cancer prevention and screening interventions in the future. First, future research needs to identify the minoritized status of a group or individual multidimensionally (eg, including economic background, location, and education) rather than unidimensionally (eg, race only). Most of the studies in this review did not address or explore the multidimensional and intersectional aspects of being minoritized, such as income level, education level, and population location, which also affect adherence to cancer screening recommendations and screening rates [57].

eHealth literacy and education level influence individuals’ self-efficacy to use interventions, such as VHAs [58]. However, the included studies rarely recruited individuals from educationally disadvantaged backgrounds. Most of the study participants had at least a high school education. Moreover, while the majority of the studies mentioned accessibility being a barrier to cancer screening, the study participants’ location or lack of health care services in their location was not mentioned, and how eHealth technology could address such barriers was not clarified. In fact, one of the most frequently cited justifications for using VHA-based interventions is geographic accessibility. However, only 4 [39,44,53,54] of the studies explicitly mentioned recruiting rural participants. Four studies [40,42,43,55] recruited urban participants only, and the rest did not mention the rural or urban location of the participants.

In addition, studies using exclusively internet-based survey platforms or electronic health records yielded a lack of diversity in their samples [38,45,46,49,54]. Studies that specifically recruited Latina farmworkers or Black men and women used community-based recruitment or a combination of internet-based and community-based recruitment [42-44,53]. One study [39] that recruited both Black and White participants and had 55% (85/154) Black participants in the sample used community-based recruitment strategies instead of internet-based recruitment [39]. Another study recruited participants in 2 phases [51]. In the first phase, it recruited participants from a senior recreation center and recruited 24/73 (33%) Black participants and 47/73 (67%) White participants. However, for the second phase of the study, they recruited through Qualtrics (Qualtrics International Inc), with 295/1400 (21%) Black participants and 1105/1400 (79%) White participants. This finding points to a need for using multipronged strategies, or community-based recruitment, when using a single strategy to ensure equitable participation from the different racial and ethnic groups.

The second recommendation to improve audience-centered VHA development is to diversify intervention languages and implementation locations. In the current review, only 1 study [55] included a population in an African, Black-majority country, and only 2 [44,55] of the 17 studies discussed interventions available in languages other than English. The final recommendation for future researchers is to measure the social presence, that is, whether users perceived the interaction with the VHAs as an interaction with another social actor (eg, health care provider) [59] of VHAs. Though VHAs have the potential to increase social presence and thus improve patient engagement in virtual settings [25], it is rarely measured or mentioned in the review studies. Hence, in the studies included, while the communication was 2-way and immediate, whether there was a perception of social presence could not be identified. Future studies can explore how much interactivity and anthropomorphous communication VHAs can provide with intended audiences, compared to traditional or noninteractive eHealth interventions (eg, pamphlets, web pages, and emails).

### Comparison With Prior Work

The current review adds to the existing literature on digital interventions for cancer prevention and screening. Recently published systematic reviews of eHealth interventions for cancer prevention and screening have focused on specific cancers (eg, cervical cancer [60,61], colorectal cancer [62], and breast cancer [63]), specific population groups (eg, Hispanic Americans [64]), or the effectiveness of the eHealth interventions (eg, increasing screening compared to usual care [65]. The current systematic review, on the other hand, has focused on eHealth interventions’ development process and identified specific strategies used for audience-centered development. In addition, previous reviews on VHA-like interactive interventions have either focused on a specific modality of the VHA (eg, animated [34], web-based interactive tools [66], chatbots [67]). The current review included a broader description of interactive digital interventions and included VHAs of any modalities available.

Although the synthesis of the included studies in the current review was inductive, the findings can be compared to existing frameworks for developing audience-centered interventions. For example, recruiting and testing the intervention on participants who are members of the intended audience is in accordance with the principles of cultural grounding, a framework for culturally sensitive intervention development [11]. The interventions in the included studies were also customized informationally, racially, and gender-wise. Customizing has been empirically proven to be an effective way to improve health outcomes, especially among minoritized groups [9]. Finally, one of the cornerstones of audience-centered intervention development is audience segmentation, that is, developing the intervention for a specific demographic rather than a generic demographic [8]. The current review identified that the studies used some type of audience segmentation by gender, race, or age.

### Strengths and Limitations

The systematic review provides a framework for audience-centered eHealth intervention development by identifying the strategies. In identifying these strategies, we extended our synthesis to “Introduction” or “Literature Review” sections of the studies, going beyond the “Methods” and “Findings” of the included studies, a standard practice in systematic reviews, but expanded the synthesis. This helped us identify rationales used to justify the usage of VHAs. Whether a new innovation addresses an existing problem needs to be evaluated to avoid techno-solutionism —the ideology that any societal issue, such as health inequities, is a discrete one and can be addressed by technology, without considering the multidimensional context (eg, sociopolitical, cultural, financial, and geographical) in which the issue is embedded [68]. The strategies identified in this review can be used as a framework for future intervention developers.

While the study systematically identified the major strategies used to develop VHAs for cancer prevention, we need to acknowledge its limitations. First, since there was no consensus in the literature about the definition of a VHA, the search terminologies included “decision aids” to ensure a more comprehensive result. However, based on the definition of VHA used in the study (2-way interaction and immediacy of communication), many of the decision aid–based studies were eliminated. This was because the description of the decision aid did not clarify whether the intervention had interactivity and immediacy of communication. Finally, the study included studies published only in the English language and in English-language journals. Hence, when making inferences or conclusions from this study, we must proceed with caution and acknowledge that this review is limited by language.

### Conclusion

This systematic review establishes an audience-centered framework for developing effective VHA interventions in cancer prevention. The identified strategies, from demographic concordance to iterative user testing, provide a blueprint for creating equitable digital health tools. However, the field now faces 3 key challenges: moving beyond Western-centric models, addressing intersectional health disparities, and developing standardized measures of VHA effectiveness. Future work must prioritize interventions that account for the complex interplay of cultural, linguistic, and structural barriers to care. By adopting this comprehensive approach, researchers can ensure VHAs fulfill their potential as transformative tools in the global effort to reduce cancer disparities through technology.

## Supplementary material

10.2196/73616 Multimedia Appendix 1 Search strategies, data extraction template, and quality appraisal details for included studies

10.2196/73616 Checklist 1 Preferred Reporting Items for Systematic Reviews and Meta-Analyses checklist.

## Abbreviations

ALEX: Agent Leveraging Empathy for eXams
MAIN: Modality, Agency, Interactivity, and Navigability
MeSH: Medical Subject Heading
PRISMA: The Preferred Reporting Items for Systematic Reviews and Meta-Analyses
VHA: Virtual Health Assistant

## References

[1] Penedo FJ, Oswald LB, Kronenfeld JP, Garcia SF, Cella D, Yanez B (2020). The increasing value of eHealth in the delivery of patient-centred cancer care. Lancet Oncol.

[2] Adunlin G, Cyrus JW, Asare M, Sabik LM (2019). Barriers and facilitators to breast and cervical cancer screening among immigrants in the United States. J Immigrant Minority Health.

[3] Black E, Hyslop F, Richmond R (2019). Barriers and facilitators to uptake of cervical cancer screening among women in Uganda: a systematic review. BMC Womens Health.

[4] Wang J, Moehring J, Stuhr S, Krug M (2013). Barriers to colorectal cancer screening in Hispanics in the United States: an integrative review. Appl Nurs Res.

[5] Sutton RT, Pincock D, Baumgart DC, Sadowski DC, Fedorak RN, Kroeker KI (2020). An overview of clinical decision support systems: benefits, risks, and strategies for success. NPJ Digit Med.

[6] Oh H, Rizo C, Enkin M, Jadad A (2005). What is eHealth (3): a systematic review of published definitions. J Med Internet Res.

[7] Atkinson NL, Gold RS (2002). The promise and challenge of eHealth interventions. Am J Health Behav.

[8] Mackert M, Mandell D, Donovan E, Walker L, Henson-García M, Bouchacourt L (2021). Mobile apps as audience-centered health communication platforms. JMIR Mhealth Uhealth.

[9] Kreuter MW, Lukwago SN, Bucholtz R, Clark EM, Sanders-Thompson V (2003). Achieving cultural appropriateness in health promotion programs: targeted and tailored approaches. Health Educ Behav.

[10] Davis RE, Resnicow K (2012). The Cultural Variance Framework for Tailoring Health Messages.

[11] Hecht ML, Krieger JLR (2006). The principle of cultural grounding in school-based substance abuse prevention: the Drug Resistance Strategies Project. J Lang Soc Psychol.

[12] Dutta MJ (2018). Culture-centered Approach in Addressing Health Disparities: Communication Infrastructures for Subaltern Voices. Commun Methods Meas.

[13] Naughton CA (2018). Patient-centered communication. Pharmacy (Basel).

[14] O’Rourke DJ, Lobchuk MM, Thompson GN, Lengyel C (2022). Expanding the conversation: a Person-centred Communication Enhancement Model. Dementia (London).

[15] Cho H (2012). Health Communication Message Design: Theory and Practice.

[16] Alpert JM, Krist AH, Aycock RA, Kreps GL (2017). Designing user-centric patient portals: clinician and patients’ uses and gratifications. Telemed J E Health.

[17] De Vito Dabbs A, Myers BA, Mc Curry KR (2009). User-centered design and interactive health technologies for patients. Comput Inform Nurs.

[18] Lauver DR, Ward SE, Heidrich SM (2002). Patient-centered interventions. Res Nurs Health.

[19] Wagner LI, Tooze JA, Hall DL (2021). Targeted eHealth intervention to reduce breast cancer survivors’ fear of recurrence: results from the FoRtitude randomized trial. J Natl Cancer Inst.

[20] Fang ML, Siden E, Korol A, Demestihas MA, Sixsmith J, Sixsmith A (2018). A scoping review exploration of the intended and unintended consequences of eHealth on older people: a health equity impact assessment. Hum Technol.

[21] Reiss K, Andersen K, Pearson E (2019). Unintended consequences of mHealth interactive voice messages promoting contraceptive use after menstrual regulation in Bangladesh: intimate partner violence results from a randomized controlled trial. Glob Health Sci Pract.

[22] Saeed SA, Masters RM (2021). Disparities in health care and the digital divide. Curr Psychiatry Rep.

[23] Chen Y, Kruahong S, Elias S (2023). Racial disparities in shared decision-making and the use of mHealth technology among adults with hypertension in the 2017-2020 health information national trends survey: cross-sectional study in the United States. J Med Internet Res.

[24] Cendan J, Lok B (2012). The use of virtual patients in medical school curricula. Adv Physiol Educ.

[25] Biocca F, Harms C, Burgoon JK (2003). Toward a more robust theory and measure of social presence: review and suggested criteria. Presence: Teleoperators Virtual Environ.

[26] Lombard M, Ditton T (2006). At the heart of it all: the concept of presence. J Comput Mediat Commun.

[27] Tudor Car L, Dhinagaran DA, Kyaw BM (2020). Conversational agents in health care: scoping review and conceptual analysis. J Med Internet Res.

[28] Robertson S, Solomon R, Riedl M, Zaphiris P, Ioannou A (2015). Learning and Collaboration Technologies.

[29] Gough D, Thomas J (2017). An Introduction to Systematic Reviews.

[30] Thomas J, O’Mara-Eves A, Harden A, Newman M (2017). An Introduction to Systematic Reviews.

[31] Thomas J, Harden A (2008). Methods for the thematic synthesis of qualitative research in systematic reviews. BMC Med Res Methodol.

[32] Page MJ, McKenzie JE, Bossuyt PM (2021). The PRISMA 2020 statement: an updated guideline for reporting systematic reviews. PLoS Med.

[33] Laranjo L, Dunn AG, Tong HL (2018). Conversational agents in healthcare: a systematic review. J Am Med Inform Assoc.

[34] Provoost S, Lau HM, Ruwaard J, Riper H (2017). Embodied conversational agents in clinical psychology: a scoping review. J Med Internet Res.

[35] About us. Covidence.

[36] Taylor SJ, Pinnock H, Epiphaniou E (2014). A Rapid Synthesis of the Evidence on Interventions Supporting Self-Management for People with Long-Term Conditions: PRISMS – Practical Systematic Review of Self-Management Support for Long-Term Conditions.

[37] Gough D, Oliver S, Thomas J (2017). An Introduction to Systematic Reviews.

[38] Mosen DM, Feldstein AC, Perrin N (2010). Automated telephone calls improved completion of fecal occult blood testing. Med Care.

[39] Vilaro MJ, Wilson-Howard DS, Neil JM (2022). A subjective culture approach to cancer prevention: rural black and white adults’ perceptions of using virtual health assistants to promote colorectal cancer screening. Health Commun.

[40] Menon U, Szalacha LA, Belue R, Rugen K, Martin KR, Kinney AY (2008). Interactive, culturally sensitive education on colorectal cancer screening. Med Care.

[41] Hong QN, Fàbregues S, Bartlett G (2018). The Mixed Methods Appraisal Tool (MMAT) version 2018 for information professionals and researchers. Educ Inf.

[42] Allen JD, Mohllajee AP, Shelton RC, Drake BF, Mars DR (2009). A computer-tailored intervention to promote informed decision making for prostate cancer screening among African American men. Am J Mens Health.

[43] Rawl SM, Skinner CS, Perkins SM (2012). Computer-delivered tailored intervention improves colon cancer screening knowledge and health beliefs of African-Americans. Health Educ Res.

[44] Wu Y, Samant D, Squibbs K, Chaet A, Morshedi B, Barnes LE (2014). Design of interactive cancer education technology for Latina farmworkers. Proc IEEE Syst Inf Eng Des Symp.

[45] Krist AH, Woolf SH, Hochheimer C (2017). Harnessing information technology to inform patients facing routine decisions: cancer screening as a test case. Ann Fam Med.

[46] Champion VL, Christy SM, Rakowski W (2018). A randomized trial to compare a tailored web-based intervention and tailored phone counseling to usual care for increasing colorectal cancer screening. Cancer Epidemiol Biomarkers Prev.

[47] Owens OL, Felder T, Tavakoli AS (2019). Evaluation of a computer-based decision aid for promoting informed prostate cancer screening decisions among African American men: iDecide. Am J Health Promot.

[48] Griffin L, Lee D, Jaisle A (2019). Creating an mhealth app for colorectal cancer screening: user-centered design approach. JMIR Hum Factors.

[49] Carter-Harris L, Comer RS, Slaven Ii JE (2020). Computer-tailored decision support tool for lung cancer screening: community-based pilot randomized controlled trial. J Med Internet Res.

[50] Vilaro MJ, Wilson-Howard DS, Griffin LN (2020). Tailoring virtual human-delivered interventions: a digital intervention promoting colorectal cancer screening for Black women. Psychooncology.

[51] Zalake M, Tavasolli F, Griffin L, Krieger J, Lok B (2021). Internet-based tailored virtual human health intervention to promote colorectal cancer screening: design guidelines from two user studies. Intell Virtual Agents.

[52] Wilson-Howard D, Vilaro MJ, Neil JM (2021). Development of a credible virtual clinician promoting colorectal cancer screening via telehealth apps for and by Black men: qualitative study. JMIR Form Res.

[53] Vilaro MJ, Wilson-Howard DS, Zalake MS (2021). Key changes to improve social presence of a virtual health assistant promoting colorectal cancer screening informed by a technology acceptance model. BMC Med Inform Decis Mak.

[54] Krieger JL, Neil JM, Duke KA (2021). A pilot study examining the efficacy of delivering colorectal cancer screening messages via virtual health assistants. Am J Prev Med.

[55] Kabukye JK, Ilozumba O, Broerse JEW, de Keizer N, Cornet R (2021). Implementation of an interactive voice response system for cancer awareness in Uganda: mixed methods study. JMIR Mhealth Uhealth.

[56] Maina IW, Belton TD, Ginzberg S, Singh A, Johnson TJ (2018). A decade of studying implicit racial/ethnic bias in healthcare providers using the implicit association test. Soc Sci Med.

[57] Domingo JLB, Chen JJ, Braun KL (2018). Colorectal cancer screening compliance among Asian and Pacific Islander Americans. J Immigr Minor Health.

[58] Bodie GD, Dutta MJ (2008). Understanding health literacy for strategic health marketing: eHealth literacy, health disparities, and the digital divide. Health Mark Q.

[59] Lee KM (2004). Presence, explicated. Commun Theory.

[60] Purohit R, Singh S, Vaishampayan D, Sane Y, Pande J, Devi S (2024). A systematic review of cervical cancer mobile applications and future directions for developers. Asian Pac J Cancer Prev.

[61] Romli R, Abd Rahman R, Chew KT, Mohd Hashim S, Mohamad EMW, Mohammed Nawi A (2022). Empirical investigation of e-health intervention in cervical cancer screening: a systematic literature review. PLoS ONE.

[62] Jiang Z, Hussain A, Grell J, Sly JR, Miller SJ (2023). Systematic review of colorectal cancer screening-related apps. Telemed J E Health.

[63] Houghton LC, Howland RE, McDonald JA (2019). Mobilizing breast cancer prevention research through smartphone apps: a systematic review of the literature. Front Public Health.

[64] Watanabe-Galloway S, Ratnapradipa K, Subramanian R (2023). Mobile health (mHealth) interventions to increase cancer screening rates in hispanic/latinx populations: a scoping review. Health Promot Pract.

[65] Richardson-Parry A, Silva M, Valderas JM, Donde S, Woodruff S, van Vugt J (2023). Interactive or tailored digital interventions to increase uptake in cervical, breast, and colorectal cancer screening to reduce health inequity: a systematic review. Eur J Cancer Prev.

[66] Kuijpers W, Groen WG, Aaronson NK, van Harten WH (2013). A systematic review of web-based interventions for patient empowerment and physical activity in chronic diseases: relevance for cancer survivors. J Med Internet Res.

[67] Wang A, Qian Z, Briggs L, Cole AP, Reis LO, Trinh QD (2023). The use of chatbots in oncological care: a narrative review. Int J Gen Med.

[68] Gardner J, Warren N (2019). Learning from deep brain stimulation: the fallacy of techno-solutionism and the need for “regimes of care”. Med Health Care Philos.

