# Supplementary material for: Virtual Health Assistants in Preventive Cancer Care Communication: Systematic Review
Source: JMIR Cancer. 2025 Sep 15;11:e73616. doi: 10.2196/73616 (PMC12435786; doi:10.2196/73616)
Supplement: Multimedia Appendix 1 [file cancer-v11-e73616-s001.docx]

**Online Supplementary Materials**

**Search Strategies**

Source: Pubmed

Search Date: 1/12/2022

Search Result: 131

Concept: VHA

Mesh: “User-Computer Interface"[Mesh] OR

Keywords: "virtual health assistant*" [tw] OR "conversational agent*" [tw] OR "virtual coach*" [tw] OR "embodied agent*" [tw] OR “avatar*” [tw] OR "relational agent*" [tw] OR "interactive agent*" [tw] OR "virtual character*" [tw] OR "animated character*" [tw] OR "virtual human*" [tw] OR “humanoid*” [tw] OR "AI agent*" [tw] OR “Chatterbox*” [tw] OR “Chatbot*” [tw] OR “Chatterbot*” [tw] OR "Chat assistant*" [tw] OR "Conversational agent*" [tw] OR "Conversational system*" [tw] OR "Conversational assistant*" [tw] OR "Conversational User Interface*" [tw] OR "Conversational interface*" [tw] OR "Conversational Character*" [tw] OR "Conversational AI*" [tw] OR "Dialog system*" [tw] OR "Interactive agent*" [tw] OR "Intelligent virtual agent*" [tw] OR "Intelligent virtual assistant*" [tw] OR "Intelligent agent*" [tw] OR “Smartbot*” [tw] OR "Talking agent*" [tw] OR "Talking avatar*" [tw] OR "Virtual personal assistant*" [tw] OR "Virtual human agent*" [tw] OR "Virtual coach*" [tw] OR "Virtual consultant*" [tw] OR "Virtual conversational agent*" [tw] OR "Virtual advisor*" [tw] OR "Virtual agent*" [tw] OR "Virtual assistant*" [tw] OR "embodied conversational agent*" [tw] OR “ECA” [tw] OR “ECAs” [tw]

Concept: Cancer

Mesh: Neoplasm

Keyword: Cancer*

Concept: Prevention

Mesh: "prevention and control" [Subheading] OR "Early Detection of Cancer"[Mesh]

Keyword: "cancer screening"[tw] OR "cancer screening test*"[tw] OR "early diagnosis of cancer"[tw]

Source: Academic Search Premier

Search Date: 12/1/2022

Search Result: 10 articles

Setting specification:

Search Modes: Boolean/Phrase;

Apply equivalent subjects

Limit Your Results: Peer reviewed

Document Type: Article

Language: English

No other criterion was specified

Search String Used:

"User-Computer Interface" OR "virtual health assistant*" OR "conversational agent*" OR "virtual coach*" OR "embodied agent*" OR “avatar*” OR "relational agent*" OR "interactive agent*" OR "virtual character*" OR "animated character*" OR "virtual human*" OR “humanoid*” OR "AI agent*" OR “Chatterbox*” OR “Chatbot*” OR “Chatterbot*” OR "Chat assistant*" OR "Conversational agent*" OR "Conversational system*" OR "Conversational assistant*" OR "Conversational User Interface*" OR "Conversational interface*" OR "Conversational Character*" OR "Conversational AI*" OR "Dialog system*" OR "Interactive agent*" OR "Intelligent virtual agent*" OR "Intelligent virtual assistant*" OR "Intelligent agent*" OR “Smartbot*” OR "Talking agent*" OR "Talking avatar*" OR "Virtual personal assistant*" OR "Virtual human agent*" OR "Virtual coach*" OR "Virtual consultant*" OR "Virtual conversational agent*" OR "Virtual advisor*" OR "Virtual agent*" OR "Virtual assistant* OR "embodied conversational agent*" OR “ECA” OR “ECAs” ) AND ( neoplasms or oncology or cancer ) AND ( "prevention and control" OR "Early Detection of Cancer" OR "cancer screening" OR "cancer screening test*" OR "early diagnosis of cancer" )

Source: Web of Science

Search Date: 1/14/2022

Search Results: 19 articles

Search String Used:

((ALL=("User-Computer Interface" OR "virtual health assistant*" OR "conversational agent*" OR "virtual coach*" OR "embodied agent*" OR “avatar*” OR "relational agent*" OR "interactive agent*" OR "virtual character*" OR "animated character*" OR "virtual human*" OR “humanoid*” OR "AI agent*" OR “Chatterbox*” OR “Chatbot*” OR “Chatterbot*” OR "Chat assistant*" OR "Conversational agent*" OR "Conversational system*" OR "Conversational assistant*" OR "Conversational User Interface*" OR "Conversational interface*" OR "Conversational Character*" OR "Conversational AI*" OR "Dialog system*" OR "Interactive agent*" OR "Intelligent virtual agent*" OR "Intelligent virtual assistant*" OR "Intelligent agent*" OR “Smartbot*” OR "Talking agent*" OR "Talking avatar*" OR "Virtual personal assistant*" OR "Virtual human agent*" OR "Virtual coach*" OR "Virtual consultant*" OR "Virtual conversational agent*" OR "Virtual advisor*" OR "Virtual agent*" OR "Virtual assistant*" OR "embodied conversational agent*" OR “ECA” OR “ECAs” )) AND ALL=("Cancer*" OR "Neoplasm*")) AND ALL=("prevention and control" OR "Early Detection of Cancer" OR "cancer screening" OR "cancer screening test*" OR "early diagnosis of cancer")

Source: EMBASE

Search Date: 1/14/2022

Search Results: 895

Search String Used, Using EmTree:

('patient'/exp OR 'patient' OR 'patients' OR 'sufferer' OR 'sufferers') AND ('decision support system'/exp OR 'decision making, computer-assisted' OR 'decision support' OR 'decision support system' OR 'decision support systems, management' OR 'decision support techniques' OR 'virtual care'/exp OR 'telehealth'/exp OR 'e-health' OR 'ehealth' OR 'tele-health' OR 'telehealth') AND ('cancer prevention'/exp OR 'cancer prevention' OR 'cancer prophylaxis' OR 'prevention, cancer' OR 'tumor prevention' OR 'tumour prevention' OR 'cancer screening'/exp OR 'cancer screening' OR 'screening, cancer' OR 'cancer prevention and control'/exp

**Data Extraction Template**

**General information**

**Study ID**

First author, year

This should constitute study ID

**Title**

Title of paper / abstract / report that data are extracted from

**Country in which the study conducted**

1. United States
2. UK
3. Canada
4. Australia
5. Other

**Cancer(s)**

please mention what cancer(s) the study focused on.

**Research question/hypothesis**

**Characteristics of included studies**

**Methods**

**Aim of study**

Copy+paste the explicitly mentioned aim/objective/purpose of the study in the abstract or introduction section of the article.

**Type of Data analyzed**

This part should be determined by the type of data being analyzed. If only numbers are analyzed- quant, only text- qual. Both- mixed. Multi-method should be chosen if one type of data (text or number) was collected in multiple ways (e.g., focus group+interviews for text, or, survey+experiment for numbers)

1. Quantitative
2. Qualitative
3. Mixed Method
4. Multi-method
5. Other

**Data collection method**

Select all that apply.

1. Interviews
2. Focus groups
3. Think alouds
4. Ethnography
5. Participant observation
6. Randomized controlled trial
7. Non-reandomized controlled trial
8. Survey
9. Participant observation
10. Not applicable
11. Other

**Participants**

**Study sample age group (Only select the group(s) that have a majority in the sample and ignore those that are minimal; select all that applies)**

age groups are borrowed from the CDC cancer patient groups mentioned here: https://www.cdc.gov/cancer/uscs/about/data-briefs/no29-USCS-highlights-2019-incidence.htm

1. 18-39
2. 40-54
3. 55-64
4. 65-74
5. 75 and over

**Study sample gender**

1. Male
2. Female
3. Both male and female
4. Not mentioned

**Study sample race**

1. Black/African American
2. White/Caucasian/European American
3. Latinx
4. Asian
5. Native American and Pacific Islanders
6. Not specified
7. Other

**Study Sample income (Select the group(s) the study sample has the majority participants of)**

Classification of the income group is made based on the information here: https://www.weforum.org/agenda/2022/07/household-income-distribution-wealth-inequality-united-states/

1. High Income (>156,000)
2. Middle Income (52,000-156,000)
3. Low Income (<52,000)
4. Not available
5. Not applicable

**Study sample location (Select the group(s) the study sample has the majority participants of)**

1. Urban
2. Rural
3. Not available
4. Not applicable

**Study sample education leve (Select the group(s) the study sample has the majority participants of)**

Sample education categories made based on the international standard classification of Education (ISCED) by UNESCO:

https://nces.ed.gov/pubs/eiip/eiip1s01.asp

1. Preprimary (Kindergarden and below)
2. Primary (grades 1-6)
3. secondary (grades 7-12)
4. Higher education (community college/university/postgraduate degrees)
5. Not available
6. Not applicable

**Inclusion criteria**

Mention what were the criteria to be eligible for participation in the study. e.g., diagnosed with cancer/ under the guidelines for screening, etc.

**Who is the VHA for/target audience/end users?**

**Study sample cancer status:**

1. cancer survivors (individuals between the diagnosis and end of life stages in cancer control contunuum)
2. Pre-cancerous/non cancerous
3. Have family history of cancer
4. Not available
5. Not applicable

**Study sample cancer screening status**

1. out of guideline
2. within guideline
3. not available
4. not applicable

**Method of recruitment of participants**

Select all that apply.

1. Phone
2. Mail
3. Clinic patients
4. Patient portal recruitment (e.g.MyChart)
5. Other

**Total number of participants**

add up all the study arms (e.g., intervention+control) if total sample is not available directly.

**VHA Development**

**VHA Characteristics**

**VHA language (select all that applies)**

1. English
2. Mandarin
3. Hindi
4. Spanish
5. French
6. Not available
7. Other

**VHA modality**

1. Text only
2. Voice only
3. Text and voice
4. visual only
5. visual and text
6. visual and voice
7. visual, voice, and text
8. Not available
9. Other

**Participant input method**

Note how human users could interact with the VHA.

1. type
2. talk
3. type and talk
4. speech to text (dictating response)
5. Not avaiable
6. Other

**VHA gender (by name)**

select all that apply

1. Male
2. Female
3. Neutral
4. Not available
5. Other

**VHA gender (by voice)**

Select all that apply

1. Male
2. Female
3. Neutral
4. Not available
5. Other

**VHA gender (by appearance)**

Select all that apply.

1. Male
2. Female
3. Neutral
4. Not available
5. Other

**VHA race**

1. Black
2. White
3. Latinx
4. Asian
5. Not available
6. Not applicable
7. Other

**VHA appearance (if available)**

if it is an animated VHA, mention the attire of the VHA, and the room/environment it is in.

If it is non embodied then, not applicable if voice-only. If it is a chatbot but has a "profile picture" of the chatbot or a thumbnail animation of the chatbot, describe that.

**Participant input capacity**

How much the participants can communicate or at what capacity the human user can communicate with the VHA

1. Can input unlimitedly (e.g., Siri or online chatbots)
2. The VHA provides limited options for the participants to choose from
3. Information not provided
4. Information unclear in the article
5. Other

**VHA Name (name of the intervention if specific name not mentioned)**

**Strategies to tailor the VHA to make it patient-centered**

**What audience needs were identified in the literature review/data?**

Audience need is defined as things that the target audience has expressed the need for regarding disease prevention (i.e., specific cancer prevention), or authors have conducted thorough literature reviews to identify the needs for. N/A in the cells if the information is not available or leave it blank.

|  | **Audience need** |
| --- | --- |
| **Need 1** |  |
| **Need 2** |  |
| **Need 3** |  |
| **Need 4** |  |
| **Need 5** |  |
| **Need 6** |  |
| **Need 7** |  |
| **Need 8** |  |
| **Need 9** |  |
| **Need 10** |  |

**How did the VHA meet those needs?**

|  | **How VHA addressed the needs** |
| --- | --- |
| **Need 1** |  |
| **Need 2** |  |
| **Need 3** |  |
| **Need 4** |  |
| **Need 5** |  |
| **Need 6** |  |
| **Need 7** |  |
| **Need 8** |  |
| **Need 9** |  |
| **Need 10** |  |

**What was the justification for using VHA, as discussed in the literature or discussion?**

**Was theory used for the development of the VHA?**

1. Yes
2. No
3. Not available
4. Not applicable
5. Other

**If yes, what theory?**

**ANy additional comments**

**Top 5 findings of the study**

|  | **Findings** |
| --- | --- |
| **1** |  |
| **2** |  |
| **3** |  |
| **4** |  |
| **5** |  |

**Quality Appraisal**

Result of quality appraisal, the Mixed Methods Appraisal Tool (MMAT)

1. Qualitative study

| Qualitative studies | Quality assessment components using MMAT | | | | | |
| --- | --- | --- | --- | --- | --- | --- |
|  | Is the qualitative approach appropriate to answer the research question? | Are the qualitative data collection methods adequate to address the research question? | Are the findings adequately derived from the data? | Is the interpretation of results sufficiently substantiated by data? | Is there coherence between qualitative data sources, collection, analysis and interpretation? | Total |
| Wu 2014 | Yes | Yes | Can’t tell | Yes | No | 60% |
| Griffin 2019 | Yes | Yes | Yes | Yes | Yes | 100% |
| Vilaro 2020 | Yes | Yes | Yes | Yes | Yes | 100% |
| Wilson-Howard 2021 | Yes | Yes | Yes | Yes | Yes | 100% |
| Vilaro 2022 | Yes | Yes | Yes | Yes | Yes | 100% |
| Total=5 |  |  |  |  |  |  |

1. Quantitative non-randomized studies

| Quantitative non-randomized studies |  | Quality assessment components using MMAT | | | | |
| --- | --- | --- | --- | --- | --- | --- |
|  | Are the participants representative of the target population? | Are measurements appropriate regarding both the outcome and intervention (or exposure)? | Are there complete outcome data? | Are the confounders accounted for in the design and analysis? | During the study period, is the intervention administered (or exposure occurred) as intended? | Total |
| Allen 2009 | No | Yes | Yes | Can’t tell | Yes | 60% |
| Owens 2019 | Yes | Yes | Yes | Yes | Yes | 100% |
| Total = 2 |  |  |  |  |  |  |

1. Quantitative descriptive studies

| Quantitative descriptive studies |  | Quality assessment components using MMAT | | | | |
| --- | --- | --- | --- | --- | --- | --- |
|  | Is the sampling strategy relevant to address the research question? | Is the sample representative of the target population? | Are the measurements appropriate? | Is the risk of nonresponse bias low? | Is the statistical analysis appropriate to answer the research question? | Total |
| Krist 2017 | Yes | Yes | Yes | Yes | Yes | 100% |
| Total = 1 |  |  |  |  |  |  |

1. Randomized control studies

| Randomized control studies | Quality assessment components using MMAT | | | | |  |
| --- | --- | --- | --- | --- | --- | --- |
|  | Is randomization appropriately performed? | Are the groups comparable at baseline? | Are there complete outcome data? | Are outcome assessors blinded to the intervention provided? | Did the participants adhere to the assigned intervention? | Total |
| Mosen 2010 | Yes | Yes | Yes | Can’t tell | Can’t tell | 60% |
| Rawl 2012 | Can’t tell | Yes | Yes | Can’t tell | Yes | 60% |
| Champion 2018 | Can’t tell | Yes | Yes | Can’t tell | Yes | 60% |
| Carter-Harris 2020 | Can’t tell | Yes | Yes | No | Can’t tell | 40% |
| Krieger 2021 | Yes | Yes | Yes | No | Can’t tell | 60% |
| Total=5 |  |  |  |  |  |  |

1. Mixed method studies

| Mixed method design | Quality assessment components using MMAT | | | | | |
| --- | --- | --- | --- | --- | --- | --- |
|  | Is there an adequate rationale for using a mixed methods design to address the research question? | Are the different components of the study effectively integrated to answer the research question? | Are the outputs of the integration of qualitative and quantitative components adequately interpreted? | Are divergences and inconsistencies between quantitative and qualitative results adequately addressed? | Do the different components of the study adhere to the quality criteria of each tradition of the methods involved? | Total |
| Menon 2008 | Yes | Yes | Yes | Can’t tell | Can’t tell | 60% |
| Vilaro 2021 | Yes | Yes | Can’t tell | Yes | Yes | 80% |
| Kabukye 2021 | Yes | Yes | No | Can’t tell | Yes | 60% |
| Zalake 2021 | Yes | Yes | Yes | Yes | Yes | 100% |
| Total = 4 |  |  |  |  |  |  |
